# Role of Circular RNAs in Preeclampsia

**DOI:** 10.1155/2019/7237495

**Published:** 2019-05-02

**Authors:** Ningyi Jia, Jian Li

**Affiliations:** Beijing Obstetrics and Gynecology Hospital, Capital Medical University, 17 QiHeLou Street, Dongcheng District, Beijing, China

## Abstract

Circular RNAs (circRNAs) are noncoding RNAs characterized by circular covalently closed structures, which are generated by back-splicing. circRNA is more stable and conserved than linear RNA and exists in various organisms. Preeclampsia (PE), a common hypertensive disorder of pregnancy, has a profound impact on maternal and neonatal mortality and morbidity. Recent studies demonstrated that circRNAs were differentially expressed in PE maternal-fetal interface compared with those in the control and might mediate pathological processes in pregnancy complications. However, the mechanisms of action of circRNAs in PE are still unclear. Here, we provide a comprehensive review on the current state of knowledge on circRNAs associated with PE. We summarize the known expression profiles of circRNAs and discuss their potential application as biomarkers of PE. The possible mechanisms underlying circRNA dysregulation in the etiology of PE are also explored.

## 1. Introduction

Noncoding RNA (ncRNA) can be classified based on their length. Small transcripts are less than 200 nucleotides and include microRNAs (miRNA) and circular RNAs (circRNAs). Long ncRNAs (lncRNAs), on the other hand, are longer than 200 nucleotides [1]. Circular RNA (circRNA) is a well-recognized, commonly found ncRNA molecule characterized by a circular covalently closed structure. They have been found to play important roles in both normal biological functioning and in the occurrence of certain diseases. Researchers have identified a number of differentially expressed circRNAs that can be used as biomarkers for the diagnosis of some diseases, such as tumors [2], neurodegenerative disorders [3], cardiovascular diseases, and injury [4].

An increasing number of studies have shown that a variety of ncRNAs are correlated with the development and progression of pregnancy-related diseases [5–9]. The purpose of this review is to discuss and highlight the biogenesis, properties, and functions of circRNAs; their potential as diagnostic markers; and their roles in the underlying pathogenesis of preeclampsia (PE).

## 2. Biogenesis of circRNA

As circRNAs have no 5′ to 3′ polarity or polyadenylated tails, they can escape degradation by RNases and are stably expressed [10–12]. Back-splicing of exons and/or introns results in the formation of circular exonic circRNAs (EcircRNAs), circular intronic RNAs (ciRNAs), and exon-intron circRNAs (EIciRNAs). These RNAs with cyclic structures are termed as circRNAs [11, 13, 14]. Jeck et al. proposed two models of exon cyclization, namely, “lariat-driven circularization” and “intron-pairing driven circularization.” The former model results in a covalent splice from the 3′ end of the splice donor to the 5′ end of the splice acceptor, leading to an exon-containing lariat structure. After the introns are removed, the lariat is formed as an exonic circle. The latter model is based on pairing of complementary motifs in the transcripts [11]. When unspecified, the term “circRNA” usually refers to the circRNAs derived from exons, in consideration of the fact that other types of circRNAs account for relatively small proportions [15, 16]. The mechanism of formation is quite different for intronic circRNA and exonic circRNA. The composition of intronic circRNA relies on the GU-rich sequences adjacent to the 5′ splice site and the C-rich sequences near the branch point. The two segments bind into a circle, and then, the spliceosome cuts out the exonic sequences and intronic sequences in the binding part. The remaining introns are eventually spliced together to form mature ciRNAs [13, 17].

ncRNAs, including circRNAs, can be protected and transported by extracellular vesicles (EVs). circRNAs are also eliminated from cells into the extracellular space via EVs. Moreover, released EVs can be taken up by other cells, indicating that the released circRNAs could contribute to cell-cell communication when taken up with the EVs [18]. The concentration of EVs in the maternal plasma increases with the progression of gestation [19]. Pregnancy-associated EVs contain protein markers, mRNAs, and miRNAs with diverse biological functions [20]. Studies demonstrate that exosomes containing miR-526b-2p can lead to PE via downregulation of HIF-1*α* and MMP-1 [21]. The interaction of different kinds of cells in the maternal-fetal interface via EVs may help us understand the physiological and pathological mechanisms of pregnancy. However, further investigations are required to clarify the role of circRNAs and EVs in PE.

## 3. Functions of circRNA

**Figure 1:**
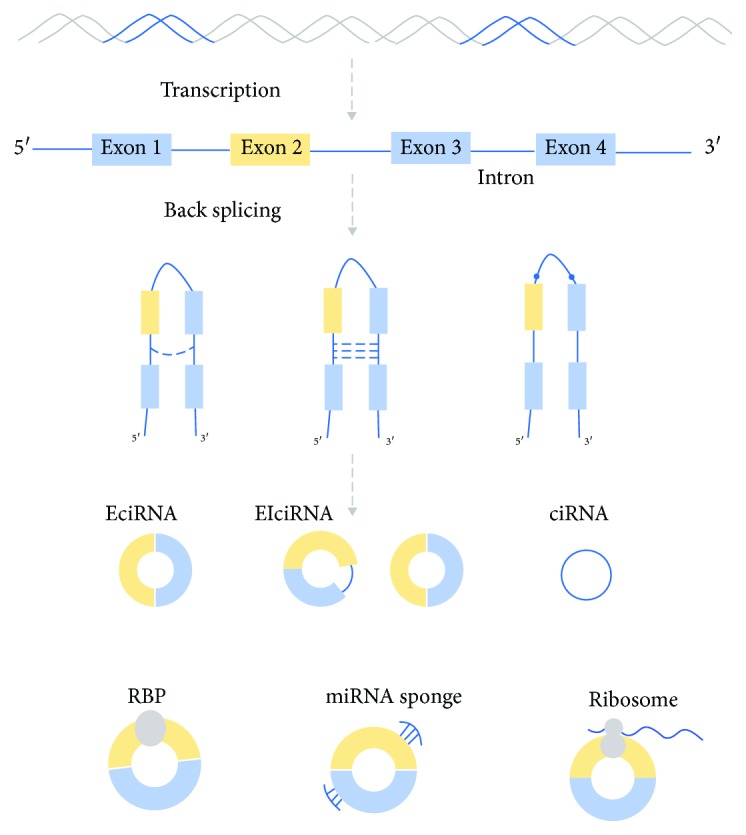
circRNAs are generated from back-splicing of exons, introns, or both, to form circular exonic circRNAs (EcircRNAs), circular intronic RNAs (ciRNAs), and exon-intron circRNAs (EIciRNAs). The functions of circRNAs include acting as microRNA (miRNA) sponges or competing endogenous RNA, interacting with RNA-binding proteins (RBPs), and regulating gene transcription and mRNA translation.

The biological function of circRNAs has been gradually recognized. However, our understanding of its functions is still rather limited when compared with that of miRNAs and lncRNAs. The majority of circRNAs is composed of EcircRNAs. EcircRNAs may function as miRNA sponges by harboring miRNA response elements (MREs). The nucleus is rich in ciRNAs and EIciRNAs, which may regulate gene transcription and posttranscriptional processing [10, 11, 13, 14, 22]. The known potential functions of circRNAs include acting as miRNA sponges or competing with endogenous RNA (ceRNA), interacting with RNA-binding proteins (RBPs), and regulating gene transcription and mRNA translation (Figure 1). These aspects are discussed in the following sections.

## 4. circRNAs Serve as miRNA Sponges or Competitors of Endogenous RNA

miRNAs, which are ncRNAs of 18-25 nucleotides, are crucial posttranscriptional regulators of gene expression [23]. miRNAs are loaded into the RNA-induced silencing complex (RISC) that recruits the target mRNA and initiates either translation inhibition or mRNA degradation [24]. In recent years, a growing number of reports have found that circRNA plays a vital role in the network of ceRNA. Other RNAs with miRNA target sites can bind miRNA and thus compete with mRNAs [25]. circRNAs have multiple MREs that allow them to compete with normal miRNA targets, resulting in weakening of the inhibitory effects of miRNA on the expression of target genes. Thus, circRNA can play a role in regulating gene expression at the transcriptional level [26].

The cerebellar degeneration-related protein 1 transcript (CDR1as), which was first reported as an miRNA sponge, has 63 binding sites for miR-7 [26]. According to the studies conducted by Memczak et al. and Hansen et al., high expression of CDR1as resulted in the downregulation of miR-7, leading to impaired midbrain development during embryogenesis and diminishing of the midbrain size in zebrafish [10, 26]. Another study on CDR1 by Xu et al. indicated that the upregulation of miR-7 in mouse islet cells contributed to insulin expression [27]. circRNA8073 can act as a ceRNA to sequester miR-181a to protect neurotensin transcripts from miR-181a-mediated suppression in endometrial epithelial cells [28]. These findings suggest that the miRNA sponge effect of circRNA may be a general phenomenon. Investigations into circRNAs are providing us with new insights to understand the etiopathogenesis of pregnancy complications as well as new potential targets for treatment.

## 5. circRNAs Interact with RBPs

Apart from interacting with miRNAs, circRNAs can also regulate gene expression and protein translation via interaction with RBPs, such as RNA polymerase II (RNA Pol II), Argonaute (AGO) proteins, muscle blind protein (MBL), and quaking I (QKI) [10, 26, 29–31], which are known to be involved in RNA editing and alternative splicing. Biogenesis of circRNAs may be mediated by flanking long introns and intronic complementary sequences. RBPs such as QKI and MBL have been associated with the biogenesis of certain circRNAs. circRNAs may be generated cotranscriptionally or posttranscriptionally [32]. Along with binding to single RBPs, circRNAs may also bind multiple RBPs and form large protein complexes via stable interactions [33]. The interaction between circRNAs and RBPs might result in an effect similar to that in the miRNA sponge, leading to depletion of RBPs, consequently reducing their interaction with RNA targets [34]. For instance, the interaction between MBL and circMBL contributes to regulation of the levels of MBL protein [30]. There is also evidence showing that QKI5 contributes to the circularization of some exons. [31]. Thus, circRNA may have the ability to regulate protein functions and protein-protein interactions [29].

## 6. circRNAs Regulate Gene Transcription and mRNA Translation

miRNA sponging and protein interactions are the known functions of circRNAs. ciRNAs can positively modulate RNA Pol II and upregulate the expression of maternal genes [35]. The EIciRNA-U1snRNA complex influences RNA Pol II to promote gene expression [14]. The highly expressed circRNAs in synapses potentially act as a medium to transport proteins and RNAs [36]. circRNAs can also be translated into proteins via a rolling circle mechanism, which provides repeated polypeptide sequences and enhances polypeptide yields per unit of time [37]. However, there is no evidence of this function of circRNAs in the reproductive system.

## 7. Role of circRNAs in PE

After 20 weeks of gestation, women with blood pressure higher than 140/90 mmHg and proteinuria are diagnosed with PE [38], a common hypertensive disorder of pregnancy affecting 3–8 % of pregnancies worldwide [39]. The symptoms of PE continue until the delivery of placenta [40]. Although the exact pathological mechanisms of PE remain unknown, it is generally believed that the dysregulation of placenta mediates pregnancy disorders. Placental abnormalities such as abnormal invasion, vasculature abnormalities, structural changes, calcification, oxidative damage, and inflammation response are closely observed in PE [41]. Several studies have suggested that dysregulated ncRNAs in the maternal-fetal interface participate in the regulation of proliferation, invasion, and apoptosis of trophoblasts, thereby promoting the pathogenesis of PE [6, 41–43]. Thus, research has focused on stable ncRNAs, which have the potential to serve as biomarkers and/or may help elucidate the pathogenesis of PE.

The potential pathophysiological mechanisms of PE may involve circRNAs. Zhou et al. demonstrated that si-circ_3286 inhibited invasion in HTR8/Svneo cells [44]. It has been known that circulating concentrations of pregnancy-associated plasma protein A (PAPP-A) were linked to the development of PE [45, 46]. Zhou et al.'s study also found that circRNA_3286 overlapped in the PAPP-A gene, suggesting that this circRNA might be associated with the pathogenesis of PE. However, these findings have not been confirmed in vivo. Another study revealed that circRNA_0001855 and circRNA_0004904 influenced the transcription of PAPP-A RNA by competing with shared microRNAs [47]. With these lines of accumulating evidence, the link between ncRNA and the pathogenesis of PE has been revealed; however, owing to the limited number of investigations and lack of in vivo research, the role of circRNA in the onset of PE remains unknown.

There are differentially expressed circRNAs in the maternal-fetal interface and in circulation in PE patients compared with those observed during normal pregnancy (Table 1). The aberrant expression of circRNAs is associated with pregnancy-related complications and pathological features [44, 47–49]. The up- and downregulated circRNAs have been analyzed by bioinformatics tools and validated by relevant experiments.

**Figure 2:**
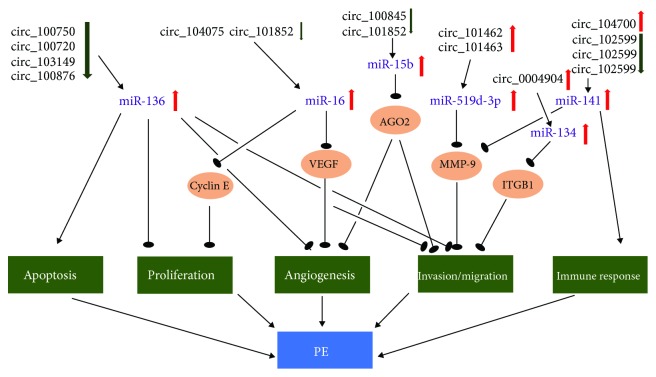
The circRNAs in maternal-interface participates in the potential pathogenesis of PE as miRNA sponge.

Although there is little evidence of a direct relation between circRNAs and PE, the correlation between miRNAs and PE has been widely established. It is reported that PE requiring termination before 34 weeks of gestation is associated with the downregulation of miR-26a-5p [50]. circRNA expression profiles showed that miR-26a-5p-relevant circ_104823, circ_104824, and circ_104819 were dysregulated in PE [48]. miR-134 is significantly upregulated in PE and negatively correlated with the expression of ITGB1. miR-134 suppresses the infiltration of trophoblast cells by targeting ITGB1 [51], while the relevant circRNAs may regulate PAPP-A by competing for shared miR-134 [47]. It was suggested that miR-16 regulates the proliferation and migration of decidua-derived mesenchymal stem cells (MSCs) by influencing VEGF expression. The overexpressed miR-16 also induces cell-cycle arrest by targeting cyclin E1. The expression level of miR-16 was negatively correlated with cyclin E1 and VEGF-A in decidua-derived MSCs in PE [52]. Ji et al. found that miR-136 significantly increased the apoptosis and inhibited the proliferation of MSCs; it could also inhibit angiogenesis and trophoblast invasion [53]. The circRNAs correlated with miR-16 and miR-136 are downregulated in PE, indicating their potential microRNA sponge function [48]. The expression of miR-141 was elevated in the PE placenta. It has been revealed that miR-141 contributes to the major function of trophoblasts and immune cells [54]. Yang et al. suggested that trophoblast cell invasion and endothelial cell tube formation were inhibited by miR-15b through downregulation of AGO2 expression [55]. The expression of miR-519d-3p was higher in the placenta from PE patients compared with that from normal pregnancies. The upregulated miR-519d-3p might inhibit the expression of MMP-9, which influences the migration and invasion of trophoblast cell [56]. circRNAs related to these miRNAs have been found to be dysregulated in PE [48, 49]. Owing to the limited number of studies on circRNA, we relate the expression profiles of circRNAs to miRNAs (Figure 2). The examples mentioned above are all based on the hypothesis that circRNAs act as miRNA sponges or ceRNAs. Further investigation is needed to confirm the relationship between circRNAs and miRNAs and their roles in the pathogenesis of PE.

Our understanding of the cellular and molecular pathogenesis of PE is still far from complete. The abnormal expression of circRNA may help us understand the pathogenic mechanism underlying PE and may act as a potential diagnostic biomarker. Considering the limited research on circRNA in pregnancy disorders, the hypothesis that circRNA plays important roles in the onset and progression of PE requires further confirmation.

## 8. Diagnostic Value of circRNAs in PE

Prediction and prevention are of great importance for pregnant women before the onset of pregnancy complications. In comparison to linear RNA, circRNAs are more stable and highly conserved in various organisms [11]. In addition, they are tissue-specific as well as developmental stage-specific [10, 11, 22]. Through EVs, circRNAs can be released to the outside of the cells [18]. Given that EVs are present in body fluids, analysis of circRNAs in EVs provides a feasible noninvasive diagnostic approach [57]. The structure of circRNAs helps them escape degradation, thereby allowing for stable expression and adding to their potential as suitable biomarkers, compared with other ncRNAs [58, 59]. Thus, differentially expressed circRNAs might be useful as noninvasive diagnostic markers for PE.

Furthermore, research indicates that circRNAs might indeed serve as potential biomarkers for PE. Jiang et al. reported circRNA expression profiling in patients with PE before the onset of symptoms. The study found that circ_0004904 and circ_0001855 combined with PAPP-A might act as biomarkers for PE detection [47]. Another study suggested that circ_0036877 could act as a ceRNA and serve as a potential biomarker for the early onset of PE [49]. The level of circ_101222 was significantly higher in PE patients' blood corpuscles than that in healthy women. In addition, combining circ_101222 and plasma protein factor endoglin may strengthen the screening efficiency [60]. These findings suggest that circRNAs might have a certain predictive value for PE.

Since circRNAs are gaining attention, future studies with a larger sample size, independent validation cohorts, or analysis of correlation with disease characteristics will help us validate the efficacy of circRNAs as biomarkers for PE. Apart from their diagnostic ability, ncRNAs could also potentially be used for the treatment of PE in the future. The shedding of ncRNAs from primary cells into the circulation may be involved in whole body cell-to-cell communication. Therefore, targeting a single ncRNA has the possibility to affect multiple downstream pathways. Recently, several different methods have been developed to silence lncRNAs in cancer [61, 62]. However, targeting multiple pathways may result in some side effects; therefore, the functions of ncRNAs (especially circRNAs discussed here) should be completely characterized before their application as therapeutic targets.

## 9. Conclusion

Benefiting from the advances of bioinformatics tools, microarray, and RNA-sequencing techniques, the role of circRNAs in the regulation of gene expression has been profoundly recognized [63]. circRNA expression profiles are examined in pregnancy complications such as gestational diabetes [64] and recurrent spontaneous abortions [65]. circRNAs have also been proved to act as important contributors in ovarian and cervical cancer [66–68]. Furthermore, emerging evidence suggests that circRNAs have great significance in pregnancy complications and gynecological cancer. However, compared with other fields, the role of circRNA in obstetrics and gynecology-related diseases is not well understood. Here, we have reviewed recent studies on circRNAs that revealed their associations with PE and put forward potential mechanisms. circRNAs could regulate gene expression and protein translation; however, recent researches focused on the function of circRNAs as miRNA sponges, such as circRNA_3286, circRNA_0001855, and circRNA_0004904 [44, 47]. Abnormally expressed circRNAs may alter the expression of certain miRNAs through binding sites, which may contribute to the development of pregnancy disorders. Besides, the types and amounts of circulating circRNAs may predict the onset of PE, including circ_0036877 and circ_101222 [49, 60]. circRNAs can also be combined with proteins for PE detection [47]. To the best of our knowledge, this is the first review that focuses on the significance of circRNAs in pregnancy disorders. Our understanding of the role of circRNAs in pregnancy complications is still at the preliminary stage. Current studies are limited to small sample sizes and lack of studies on the in vivo mechanism. circRNAs appear to be dysregulated in the pathological state and are speculated to serve as diagnostic biomarkers for PE, given their special structure, high stability, differential expression, and involvement in placental development. In future studies, it will be of great significance to identify more circRNAs involved in pregnancy disorders and explore their functions and targets.

## Figures and Tables

**Table 1:** circRNA expression profiles in pregnancy complications.

Reference	Sample	Pregnancy complication	Filtering criteria	Method	Number of upregulated circRNAs	Number of downregulated circRNAs
[44]	Placenta	PE	FC > 2.0; *p* value < 0.05	RNA-seq & qRT-PCR	2	47
[47]	Whole blood	PE	FC > 2.0; *p* value < 0.05	Microarray & qRT-PCR	1294	884
[48]	Placenta	PE	FC ≥ 2.0; *p* value ≤ 0.05	Microarray & qRT-PCR	143	158
[49]	Whole blood	PE	FC > 2.0 or <0.5; *p* value < 0.05	Microarray & qRT-PCR	4569	3984

FC: fold change; PE: preeclampsia; qRT-PCR: real-time quantitative PCR.
